# Drugging Ras trafficking—are there new roads to travel?

**DOI:** 10.1038/s44386-025-00012-7

**Published:** 2025-06-04

**Authors:** Elisabeth Schaffner-Reckinger, Atanasio Gómez-Mulas, Daniel Kwaku Abankwa

**Affiliations:** Cancer Cell Biology and Drug Discovery Group, Department of Life Sciences and Medicine, University of Luxembourg, Esch-sur-Alzette, Luxembourg

**Keywords:** Drug discovery, Target validation

## Abstract

Inhibition of Ras trafficking was the first approach to target Ras in the clinic. With the advent of direct Ras inhibitors, trafficking inhibition may appear obsolete. However, targeting certain trafficking hubs may still offer unexpected opportunities. To exploit these, we need to learn more about the functioning of Ras in specific organelles, which are associated with e.g., cell differentiation. We here discuss future opportunities in that regard.

## Introduction

**Fig. 1:**
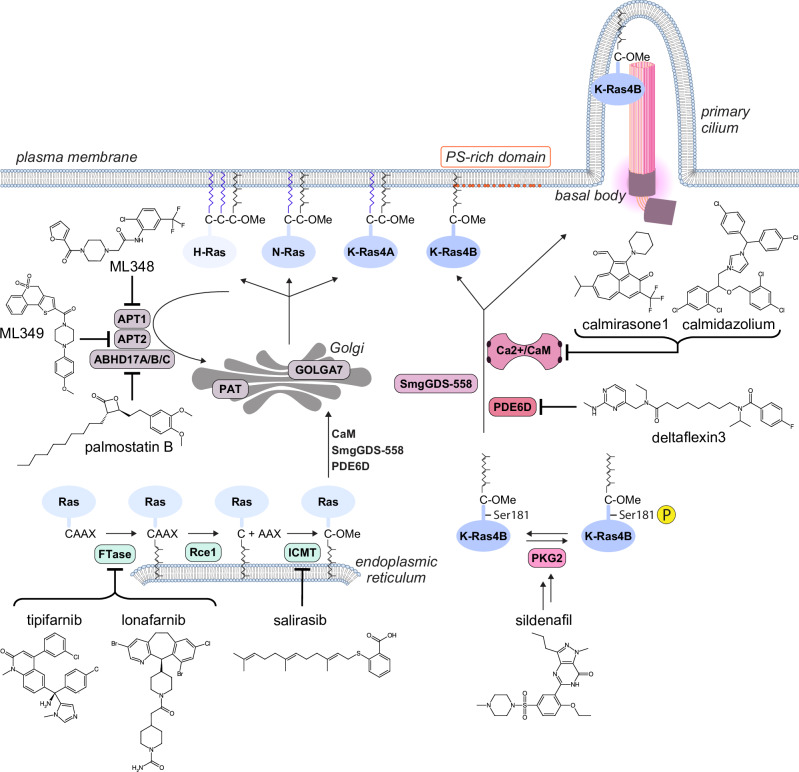
Schematic overview of selected drugs targeting Ras lipidation and trafficking. Example compounds that inhibit or modulate indicated targets are shown with their names. Several are approved or clinical-stage drugs (all compounds in the bottom row). The left-hand side depicts lipid modification pathways of Ras, while the right side shows the action of trafficking chaperones of Ras. On the endoplasmic reticulum, all Ras proteins are initially farnesylated on the C-terminal cysteine of the CAAX-box (C: cysteine, A: aliphatic residue, X: any residue) followed by subsequent RCE1-mediated AAX-tripeptide cleavage and carboxymethylation of the cysteine by ICMT (enzymes shown in turquoise). Subsequently, K-Ras4B becomes trapped on the recycling endosome (not shown), while the other Ras isoforms are trapped on the Golgi-apparatus by palmitoylation. Enzymes mediating re- and depalmitoylation are shown in gray hues. All Ras are subsequently vesicularly transported to the plasma membrane, where they laterally segregate into distinct nanoclusters. Phosphatidylserine (PS) has emerged as an important lipid to mediate this process by directly interacting with the most C-terminal residues of Ras proteins. Extraction or dissociation from the plasma membrane is enhanced after depalmitoylation. In addition, endocytosis may return Ras into the cycle for vesicular transport. Trafficking chaperones (purple hues) solubilize Ras whenever it has to move between membranes by binding to the farnesyl moiety thus allowing its long-range diffusion. This is necessary to fuel the transport cycle to the plasma membrane and other organelles. Given the distinct client spectrum and their potential involvement in specific cell- and developmental pathways, trafficking chaperones may offer more discrete drug targeting opportunities than broad inhibition of the lipidation machinery.

The small GTPase Ras operates as a membrane-anchored switchable recruitment site for downstream effectors. Given that membrane anchorage is indispensable, the first Ras drugs evaluated in the clinic were farnesyltransferase inhibitors, which block the attachment of the first obligate lipid modification on the C-terminus of Ras (Fig. 1). In the early 2000s, both inhibitors tipifarnib and lonafarnib failed and Ras was deemed undruggable. At that time, it was underappreciated that the proteins encoded by the most frequently mutated *RAS* gene *KRAS*, K-Ras4A and K-Ras4B, and N-Ras can be alternatively prenylated with a geranylgeranyl-moiety^1^.

Fortunately, the inhibitors that were developed during this period experienced a late renaissance. Lonafarnib was found to be a well-tolerated, FDA-approved treatment for the accelerated aging disease Hutchinson-Gilford progeria syndrome, and tipifarnib was FDA-approved in 2021 for the treatment of head and neck cancers with mutated *HRAS*^2,3^.

In this perspective, we mostly focus on K-Ras, due to its high mutation frequency in cancer and the possibility to target it directly^4^. There are now two approved direct K-Ras-G12C inhibitors in the clinic^5^, but also a deluge of direct Ras inhibitors under development, which promise even greater success. As compared to approved OFF-state inhibitors, Ras (ON)-state inhibitors, target the predominant, GTP-loaded active conformation of oncogenic Ras^6^ (Table S1). The reversible pan-K-Ras (OFF) inhibitor BI-2865 can block wild-type and several oncogenic *KRAS* alleles^7^. An even broader activity is expected with the reversible tri-complex Ras (ON)-multi-selective inhibitor RMC-6236, which targets multiple wildtype and oncogenic Ras isoforms and remains potent in K-Ras-G12C inhibitor-resistant preclinical models^8^. All these new inhibitors have the potential to overcome some or several limitations associated with the first generation of K-Ras-G12C inhibitors. Excitingly, recent work further suggests that the cryptic switch II pocket is also targetable in other Ras- and Rho-family members, suggesting an enormous potential to broadly target small GTPases in the future by capitalizing on the chemical scaffolds established with covalent K-Ras-G12C inhibitors^9^. This opportunity conversely indicates a liability, as inhibitors with this scaffold may spuriously target multiple GTPases or non-mutant Ras isoforms leading to side effects.

An obvious way to increase clinical efficacy and circumvent the occurrence of resistance is to apply drug combination regimes^10^. However, ideally, one would want to target an activity of oncogenic Ras that drives the emergence of resistant clones. Our current understanding of Ras is that it relays mitogenic stimuli into the cell and drives cell cycle progression and proliferation. Thus, it would seem plausible that oncogenic Ras-transformed clones have a proliferative advantage to outcompete other clones. This concept is implied in countless depictions of the Ras pathway topography, where the signal input at the top (mitogen) has a matched output at the bottom (proliferation). This simplifying pathway thinking, which predominates our current “systems-level” view, has its roots in genetic experiments performed in model organisms. Genetic dependency experiments could tell if a gene was up- or downstream of another. However, typically, these statements were limited to a certain developmental and/or tissue context.

How can we get back to the tissue and developmental stage-specific context of signaling events in Ras and cancer research? The role of Ras isoforms during development and cell differentiation is only poorly understood. Scant evidence suggests that the differentiation of cells and self-renewal of progenitors depend on specific Ras isoforms H-Ras, N-Ras, K-Ras4A, or K-Ras4B^11,12^. This is remarkable, as it points to Ras being involved in a fundamental process of metazoan life.

Given that several mouse models have already investigated the overall developmental impact of these Ras isoforms, their specific role in cell differentiation in simple cellular models may appear less noteworthy. However, the impressions obtained with Ras mouse models are not exactly coherent. On the one hand, knockouts of *HRAS* and/or *NRAS* are viable and were regarded as largely normal^13,14^, while on the other hand, recent data show that surviving double-knockouts of these two genes display RASopathy phenotypes^15^. It is surprising that single knockouts appear normal, while the double knockout shows a disease phenotype. This may suggest that RASopathies can also be associated with the loss of certain Ras isoforms, such as in the double knockout. Indeed, several RASopathy *KRAS* mutants carry also loss-of-function features, such as reduced effector binding^16^. Thus, MAPK-pathway activity is only slightly, if at all, elevated as compared to oncogenic hotspot *RAS* mutants. These are typically not seen in RASopathies, with the exception of *HRAS-G12S*-associated Costello syndrome. Its transgenic mouse model is driven by an *HRAS-G12V* mutation and displays grossly abnormal developmental defects similar to what is seen in human patients^17^. Given that *HRAS* mutations cause Costello syndrome in humans, while mutations in *KRAS* or *NRAS* are associated with the RASopathy Noonan syndrome, it appears that the type of developmental syndrome is also associated with specific Ras isoforms^18^.

Understanding the developmental activities of Ras requires the longitudinal examination of Ras activity and, probably, the study of signaling events localized to subcellular structures as compared to averaged signals in the bulk of cell lines or tissues^11,19^. On the flip side, targeting Ras trafficking to these subcellular structures may become again more relevant in drug development.

In this perspective, we will keep these big questions of the role of Ras isoforms during normal and abnormal development in mind when reflecting on untapped drug targeting opportunities in Ras trafficking pathways. For comprehensive recent reviews of Ras trafficking and membrane organization and its drug targeting potential en route to the plasma membrane, we refer to recent reviews^20,21^.

## Ras lipid domains of the Golgi apparatus and the plasma membrane may offer new targeting opportunities

The cancer-associated Ras isoforms differ mostly in their last ~20 C-terminal residues and their engagement of the membrane via their allosteric lobe (residues 87-171), which largely determine their subcellular trafficking and lateral organization in plasma membrane lipid domains^20,22^. Therefore, trafficking and membrane localization must carry a significant amount of biological information that explains isoform differences. It is undisputed that Ras executes its major activity at the plasma membrane, where mitogen-sensing receptors are embedded. Ras can subsequently redistribute to many membranous organelles of the cell, whether it is internalized by endocytosis or detached by dissociation^23,24^. Targeting of Ras endocytosis may still hold drug development potential and deserves further investigation in the future.

Interference with both the de- and repalmitoylation cycles of Ras was shown to delocalize H-Ras, N-Ras, and K-Ras4A in cells^24,25^. Both H-Ras and N-Ras are trapped on the Golgi apparatus for S-palmitoylation and subsequent vesicular transport to the plasma membrane^26^. K-Ras4A is found on mitochondrial membranes when depalmitoylated, where it directly binds and activates hexokinase I as a novel effector^27^.

Inhibition of protein palmitoyltransferase activity has so far proven difficult^28^. The most commonly used compound, 2-bromopalmitate, is overall non-selective, blocking the formation of acylase intermediates and fatty acid biosynthesis. Interestingly, inhibition of depalmitoylating enzymes acyl protein thioesterases APT1 and α/β hydrolase domain 17 (ABHD17) by palmostatin B led to Ras being redistributed to internal membranes^29,30^. Remarkably, the APT1- and APT2-specific inhibitors ML348 and ML349, respectively, or knockdown of their targets does not inhibit the growth of *NRAS* mutant melanoma cells^31^. By contrast, palmostatin B does, suggesting that inhibition of ABHD17 is more relevant in this context (Fig. 1, left).

The dually palmitoylated Golgi-resident protein golgin subfamily A number 7 (GOLGA7) stabilizes the N-Ras and H-Ras protein acyltransferase ZDHHC9. Its knockout however led to the mislocalization specifically of N-Ras from the plasma membrane^32^. This might point to N-Ras and H-Ras being palmitoylated in distinct compartments of the Golgi. Targeting of such compartments may open new isoform-specific drug targeting opportunities.

Targeting the lateral segregation of Ras proteins into plasma membrane nanoclusters represents another untapped opportunity to block specific Ras isoforms. Ras nanoclusters are proteo-lipid assemblies of di-/oligomeric Ras, which serve as platforms for effector recruitment^33^. The recent years have seen great progress in our understanding of the lipid specificity that is encoded in the C-terminus of Ras. The polybasic lysine stretch of K-Ras4B does not electrostatically anchor the protein to the membrane, but instead adopts an ensemble of distinct conformations that mediate lipid recognition, in particular of phosphatidylserine (PS)^34,35^. PS is thereby important to maintain the proper lateral segregation between H-Ras and K-Ras4B and maintain nanoclusters of K-Ras4B^20^. Interference with PS trafficking by fendiline treatment reduced K-Ras4B nanoclustering, cancer cell proliferation, and tumor growth in vivo^36^. Likewise, ablation of PS transport proteins ORP5 and ORP8 significantly blocked growth of pancreatic cancer cell xenografts^37^. The Hancock group furthermore discovered that metabolic reprogramming by oncogenic *KRAS* alters a subset of glycosphingolipids on the outer leaflet. One of these, GM3, couples through the lipid bilayer to maintain PS on the inner leaflet and thus K-Ras4B nanoclustering^38^. Targeting of this intricate feedforward loop blocked pancreatic cancer cell growth in vitro and in vivo. However, with the inhibition of enzymes responsible for Ras lipidation or of lipids of specific membrane domains, a broad effect on the trafficking and membrane organization of many other proteins can be expected and may contribute to undesired biological effects.

Different oncogenic mutants of K-Ras4B display distinct conformational dynamics, leading to lipid headgroup and acyl chain-specific engagements of the G-domain with the plasma membrane^39^. By using a covalent switch II pocket inhibitor that was modified with a long, lipophilic acyl chain, it could be demonstrated that restraining the plasma membrane orientation of K-Ras4B-G12C with such a compound disrupts its nanoclustering more potently than the parental MRTX849^40^. Therefore, taking the conformational dynamics of oncogenic Ras in its native membrane environment into account may present novel, more specific inhibitor design opportunities.

## Targeting Ras prenylation and prenyl-binding chaperones

As mentioned at the outset, the historically first approach to target Ras was to inhibit farnesyltransferase, which culminated in the development of tipifarnib and lonafarnib. Once it was recognized that K-Ras and N-Ras become geranylgeranylated upon inhibition of farnesyltransferase, CAAX-mimetic inhibitors of geranylgeranyltransferase I and dual inhibitors targeting both prenyltransferases were developed. While GGTI-2418 progressed into the clinic, it was soon recognized that dual inhibition of farnesyltransferase and geranylgeranyltransferase was too toxic^41^.

Likewise, inhibition of other CAAX-processing enzymes, isoprenylcysteine carboxylmethyltransferase (ICMT) and Ras converting enzyme 1 (RCE1), may be too toxic due to their broad impact^1^. Relatively little progress was made in advancing inhibitors of these enzymes to the clinic, with the exception of salirasib, which inhibits ICMT with micromolar (Ki = 2.6 μM) potency^42,43^.

Three prenyl-binding trafficking chaperones have been identified, which solubilize the hydrophobic prenyl-moiety of several Ras proteins to allow for their long-range diffusion through the cytoplasm, thus feeding the cycle that transports Ras proteins to the plasma membrane^44^. These chaperones, SmgGDS-558, calmodulin (CaM), and PDE6D, promiscuously facilitate solubilization also of other small GTPases^45–47^. CaM in addition has the ability to extract K-Ras4B from the membrane^47,48^. Furthermore, the retromer coat protein VPS35 emerged from a proteomics screen as a specific trafficking chaperone of farnesylated N-Ras^49^. All these chaperones bind primarily the prenyl moiety of small GTPases, with some contribution of polybasic residues at the C-terminus of the cargo to CaM and SmgGDS-558 binding. Therefore, these chaperones generally do not discriminate between the active or inactive conformation of Ras. However, specific contacts between cargo and chaperone define distinct cargo specificities^45,46,50–52^. In general, palmitoylation close to the prenylated cysteine blocks binding to these chaperones. By contrast, carboxymethylation of the farnesylated cysteine increases the affinity of cargo proteins to the trafficking chaperone PDE6D^50^. Therefore, inhibitors of ICMT can be predicted to act synergistically with PDE6D inhibitors. Such an activity may be combined with the ICMT inhibitor salirasib, which, as a farnesyl-derivative, also seems to inhibit PDE6D (DA unpublished observations). This combination opportunity may kindle novel interest in the development of ICMT inhibitors.

Phosphorylation of Ser181 by PKC or PKG2 at the C-terminus of K-Ras4B reduces its affinity to both CaM and PDE6D^50,53,54^. This illustrates an interesting selectivity opportunity to inhibit the binding preferentially of only those few small GTPases to these chaperones, which can be phosphorylated at their C-terminus in a similar manner as K-Ras4B^55^. In combination with incomplete chaperone inhibition, this could limit the expected broad impact of full chaperone inhibition to potentially only a few small GTPases. Moreover, it is plausible to assume that the three trafficking chaperones have significantly overlapping functions. However, there may be (patho)biological conditions that are still insufficiently understood, where the role of one or the other chaperone predominates. Therefore, targeting of trafficking chaperones offers a more focused opportunity for the inhibition of prenylated proteins, as compared to the inhibition of prenyltransferases (Fig. 1, right).

Since the 1980s, several CaM inhibitors have been developed, which naturally target multiple activities of CaM beyond those of chaperoning Ras. CaM inhibiting neuroleptic phenothiazines at some stage even advanced into the clinic, given the involvement of CaM in cell proliferation^56^. The most potent non-covalent CaM inhibitor is calmidazolium^57^. The natural product ophiobolin A is a potent covalent inhibitor of CaM, but also reacts with several other proteins, explaining its broad toxicity. Recently a less toxic and more selective synthetic analog, calmirasone1, was identified, which more selectively reduces K-Ras4B membrane association^58^. Interestingly, these inhibitors also target the interaction of K-Ras4B with centrin1, a protein highly related to CaM^59^. Surprisingly, oncogenic K-Ras4B mutants show a higher engagement with both proteins, suggesting complexation in concert with effectors^58–61^. At the same time, centrin1 depletion has a minor effect on K-Ras4B membrane anchorage. Thus, CaM more so than centrin1 may act as a trafficking chaperone in cells, while in addition being in complex with active K-Ras4B and effectors^59^. It can be speculated that such complexes are formed during mitosis and cytokinesis when CaM localizes to the plasma membrane and both CaM and centrin1 are found on centrosomes^59,62,63^. At both locations, these chaperones may thus likewise serve as transient docking sites of K-Ras4B. However, a function of centrosomal K-Ras4B is not described.

The other major Ras trafficking chaperone, PDE6D was nominated as a surrogate target of K-Ras4B. This led to the development of multiple PDE6D inhibitors, notably by the Waldmann group^64,65^ (Table S2). Shortcomings of these inhibitors included off-target effects and poor solubility. The latter may seem inevitable when raising inhibitors against a highly hydrophobic pocket. While the recently developed inhibitor Deltaflexin3 overcomes these issues, it shares the relatively poor inhibitory activity on MAPK-signaling with the other compounds^55^. By combining it with the approved drug Sildenafil, which facilitates the phosphorylation of K-Ras4B Ser181 and thus lowers its PDE6D affinity, the inhibitory effect could be increased but remained modest. This is most easily rationalized by accepting that this trafficking chaperone contributes only to an estimated 25-50% of K-Ras4B membrane trafficking^55^. A way forward in PDE6D inhibitor development may be inspired by the fact that natural cargo modulates its affinity to the chaperone at the entrance of the prenyl-binding pocket^51^.

## A new perspective on how to target Ras trafficking in diseases

The natural cargos of PDE6D include many proteins destined for the primary cilium, a crucial antenna-like organelle on the surface of stem- and progenitor-cells, which senses developmental signaling cues^51^. The cilium emerges from the basal body, which originates from the mother centrosome in resting cells. K-Ras4B has been detected inside the cilium, in line with the fact that PDE6D-cargo release factor Arl3 is active in the cilium and can facilitate ejection of both high- and low-affinity cargo, such as K-Ras4B into the cilium^66,67^. However, in another instance only a mutant with increased PDE6D affinity was detected in the cilium, while the wild-type K-Ras4B was not^68^.

It is interesting to note that both PDE6D and CaM are associated with the two centriolar organelles, the primary cilium and centrosome. Both organelles are not only involved in cell division but have distinctive roles during cell differentiation, where inheritance of the mother centrosome and the remnant of the primary cilium protect stemness in the inheriting daughter cell^69^. Importantly, this affords a general mechanism for asymmetric cell divisions, a process that is critical as tissues need to regenerate in the adult organism.

The profound importance of PDE6D during development is highlighted by the fact that its germline loss-of-function mutations are associated with the ciliopathy Joubert-Syndrome, which affects multiple organ systems^70^. By contrast, CaM is not directly associated with a developmental disease, which can be explained by the fact that humans have three gene copies of it, CALM1-3. Instead, CaM is associated with the “ADORA2B mediated anti-inflammatory cytokine production” SuperPath (https://tinyurl.com/2d2wapxc), which incidentally is associated with several RASopathies (https://tinyurl.com/3s9dx3a5). ADORA2B is an adenosine-activated G-protein coupled receptor that can influence the Ras-pathway via its second messenger cAMP and is negatively associated with cancer survival, while its activation is positively associated with axon regeneration in mouse retinal ganglion cells^71,72^. Indeed, ciliopathies and RASopathies share several of their multi-organ phenotypic defects, including musculoskeletal and neurodevelopmental defects, and some RASopathies exhibit loss of cilia in animal models^73^.

Could this mean that Ras proteins have a role in the functioning of the primary cilium? It would place Ras at the center of a stem- and progenitor-cell resident organelle that regulates major developmental decisions, which are not only important during organismal development but also during repair in the adult^74^.

Cancer has often been described as ‘the wound that never heals’, i.e. an incomplete repair process. In this context, it is interesting to note that oncogenic Ras transformation alone can significantly block terminal cell differentiation^11,75,76^. This process would not only keep cells in a less differentiated state but also allow them to continue proliferating slowly, thus imparting two major hallmarks observed in cancer. Implicitly, this would mean that transformation by Ras alone sustains clones that continue to proliferate and are not fully differentiated and immaturity traits that could appear as “stem-like” such as seen in cancer stem cells^19^. Moreover, Ras-transformed cells and cancer cells typically do not form a primary cilium, thus transformation also subtracts from the pool of tissue-resident stem-/progenitor cells that would promote normal repair.

Given our currently still insufficient knowledge about the details of the underlying cellular and molecular processes, it remains difficult to predict if inhibition, in particular of K-Ras4B trafficking to the cilium or the centrosome, would be beneficial in Ras-driven diseases. However, it is clear that targeting K-Ras4B trafficking may not only affect processes associated with plasma membrane-bound K-Ras4B but also those (unknown) processes tied to other organelles. Hence, the microscopic and mesoscopic spatio-temporal context, i.e. inside the cell during the cell cycle, or in tissues during development, should probably become part of the consideration of how to target K-Ras4B (trafficking) in diseases. By combining Ras trafficking inhibitors with currently developed direct Ras-inhibitors, one may not only identify novel synergistic activities but possibly also enable fine tuning of the total inhibitory activity.

We conclude that inhibition of specific K-Ras4B trafficking routes still holds some potential. To enable this, we advocate to reexamine the trafficking-dependent mechanisms underlying the disease-associated differentiation activities of Ras. In the foreseeable future, however, direct Ras inhibitors will dominate the development of therapies against aberrant Ras activities.

## Supplementary information

SI Tables

## Data Availability

No datasets were generated or analyzed during the current study.

## References

[1] Berndt, N., Hamilton, A. D. & Sebti, S. M. Targeting protein prenylation for cancer therapy. *Nat. Rev. Cancer* **11**, 775–791 (2011). 22020205 10.1038/nrc3151 PMC4037130

[2] Suzuki, M. et al. FDA approval summary for lonafarnib (Zokinvy) for the treatment of Hutchinson-Gilford progeria syndrome and processing-deficient progeroid laminopathies. *Genet Med.* **25**, 100335 (2023). 36507973 10.1016/j.gim.2022.11.003

[3] Ho, A. L. et al. Tipifarnib in head and neck squamous cell carcinoma With HRAS mutations. *J. Clin. Oncol.* **39**, 1856–1864 (2021). 33750196 10.1200/JCO.20.02903 PMC8189627

[4] Punekar, S. R., Velcheti, V., Neel, B. G. & Wong, K. K. The current state of the art and future trends in RAS-targeted cancer therapies. *Nat. Rev. Clin. Oncol.* **19**, 637–655 (2022). 36028717 10.1038/s41571-022-00671-9 PMC9412785

[5] Steffen, C. L., Kaya, P., Schaffner-Reckinger, E. & Abankwa, D. Eliminating oncogenic RAS: back to the future at the drawing board. *Biochem. Soc. Trans.* **51**, 447–456 (2023). 36688434 10.1042/BST20221343 PMC9987992

[6] Sharma, A. K. et al. Revealing the mechanism of action of a first-in-class covalent inhibitor of KRASG12C (ON) and other functional properties of oncogenic KRAS by (31)P NMR. *J. Biol. Chem.* **300**, 105650 (2024). 38237681 10.1016/j.jbc.2024.105650 PMC10877953

[7] Kim, D. et al. Pan-KRAS inhibitor disables oncogenic signalling and tumour growth. *Nature* **619**, 160–166 (2023). 37258666 10.1038/s41586-023-06123-3 PMC10322706

[8] Holderfield, M. et al. Concurrent inhibition of oncogenic and wild-type RAS-GTP for cancer therapy. *Nature* **629**, 919–926 (2024). 38589574 10.1038/s41586-024-07205-6 PMC11111408

[9] Morstein, J. et al. Targeting Ras-, Rho-, and Rab-family GTPases via a conserved cryptic pocket. *Cell*, 10.1016/j.cell.2024.08.017 (2024). 10.1016/j.cell.2024.08.017 PMC11531380 39255801

[10] Sattler, M., Mohanty, A., Kulkarni, P. & Salgia, R. Precision oncology provides opportunities for targeting KRAS-inhibitor resistance. *Trends Cancer* **9**, 42–54 (2023). 36751115 10.1016/j.trecan.2022.10.001

[11] Chippalkatti, R. et al. RAS isoform-specific activities are disrupted by disease-associated mutations during cell differentiation. *Eur. J. Cell Biol.* **103**, 151425 (2024). 38795504 10.1016/j.ejcb.2024.151425

[12] Quinlan, M. P., Quatela, S. E., Philips, M. R. & Settleman, J. Activated Kras, but not Hras or Nras, may initiate tumors of endodermal origin via stem cell expansion. *Mol. Cell Biol.* **28**, 2659–2674 (2008). 18268007 10.1128/MCB.01661-07 PMC2293097

[13] Malumbres, M. & Barbacid, M. RAS oncogenes: the first 30 years. *Nat. Rev. Cancer* **3**, 459–465 (2003). 12778136 10.1038/nrc1097

[14] Esteban, L. M. et al. Targeted genomic disruption of H-ras and N-ras, individually or in combination, reveals the dispensability of both loci for mouse growth and development. *Mol. Cell Biol.* **21**, 1444–1452 (2001). 11238881 10.1128/MCB.21.5.1444-1452.2001 PMC86690

[15] Fuentes-Mateos, R. et al. Combined HRAS and NRAS ablation induces a RASopathy phenotype in mice. *Cell Commun. Signal* **22**, 332 (2024). 38886790 10.1186/s12964-024-01717-4 PMC11184836

[16] Gremer, L. et al. Germline KRAS mutations cause aberrant biochemical and physical properties leading to developmental disorders. *Hum. Mutat.* **32**, 33–43 (2011). 20949621 10.1002/humu.21377 PMC3117284

[17] Schuhmacher, A. J. et al. A mouse model for Costello syndrome reveals an Ang II-mediated hypertensive condition. *J. Clin. Investig.* **118**, 2169–2179 (2008). 18483625 10.1172/JCI34385 PMC2381749

[18] Rauen, K. A. The RASopathies. *Annu Rev. Genom. Hum. Genet* **14**, 355–369 (2013). 10.1146/annurev-genom-091212-153523 PMC4115674 23875798

[19] Chippalkatti, R. & Abankwa, D. Promotion of cancer cell stemness by Ras. *Biochem. Soc. Trans.* **49**, 467–476 (2021). 33544116 10.1042/BST20200964 PMC7925005

[20] Zhou, Y., Gorfe, A. A. & Hancock, J. F. RAS nanoclusters selectively sort distinct lipid headgroups and acyl chains. *Front. Mol. Biosci.* **8**, 686338 (2021). 34222339 10.3389/fmolb.2021.686338 PMC8245699

[21] Pavic, K., Chippalkatti, R. & Abankwa, D. Drug targeting opportunities en route to Ras nanoclusters. *Adv. Cancer Res.* **153**, 63–99 (2022). 35101236 10.1016/bs.acr.2021.07.005

[22] Abankwa, D., Gorfe, A. A., Inder, K. & Hancock, J. F. Ras membrane orientation and nanodomain localization generate isoform diversity. *Proc. Natl. Acad. Sci. USA* **107**, 1130–1135 (2010). 20080631 10.1073/pnas.0903907107 PMC2824305

[23] Schmick, M. et al. KRas localizes to the plasma membrane by spatial cycles of solubilization, trapping and vesicular transport. *Cell* **157**, 459–471 (2014). 24725411 10.1016/j.cell.2014.02.051

[24] Rocks, O. et al. The palmitoylation machinery is a spatially organizing system for peripheral membrane proteins. *Cell* **141**, 458–471 (2010). 20416930 10.1016/j.cell.2010.04.007

[25] Tsai, F. D. et al. K-Ras4A splice variant is widely expressed in cancer and uses a hybrid membrane-targeting motif. *Proc. Natl. Acad. Sci. USA* **112**, 779–784 (2015). 25561545 10.1073/pnas.1412811112 PMC4311840

[26] Rocks, O. et al. An acylation cycle regulates localization and activity of palmitoylated Ras isoforms. *Science* **307**, 1746–1752 (2005). 15705808 10.1126/science.1105654

[27] Amendola, C. R. et al. KRAS4A directly regulates hexokinase 1. *Nature* **576**, 482–486 (2019). 31827279 10.1038/s41586-019-1832-9 PMC6923592

[28] Hu, X. et al. A mini review of small-molecule inhibitors targeting palmitoyltransferases. *Eur. J. Med. Chem. Rep.* **5**, 100041 (2022).

[29] Dekker, F. J. et al. Small-molecule inhibition of APT1 affects Ras localization and signaling. *Nat. Chem. Biol.* **6**, 449–456 (2010). 20418879 10.1038/nchembio.362

[30] Lin, D. T. & Conibear, E. ABHD17 proteins are novel protein depalmitoylases that regulate N-Ras palmitate turnover and subcellular localization. *Elife* **4**, e11306 (2015). 26701913 10.7554/eLife.11306 PMC4755737

[31] Vujic, I. et al. Acyl protein thioesterase 1 and 2 (APT-1, APT-2) inhibitors palmostatin B, ML348 and ML349 have different effects on NRAS mutant melanoma cells. *Oncotarget* **7**, 7297–7306 (2016). 26771141 10.18632/oncotarget.6907 PMC4872786

[32] Liu, C. et al. GOLGA7 is essential for NRAS trafficking from the Golgi to the plasma membrane but not for its palmitoylation. *Cell Commun. Signal* **22**, 98 (2024). 38317235 10.1186/s12964-024-01498-w PMC10845536

[33] Abankwa, D. & Gorfe, A. A. Mechanisms of Ras membrane organization and signaling: Ras rocks again. *Biomolecules* **10**, 10.3390/biom10111522 (2020). 10.3390/biom10111522 PMC7694788 33172116

[34] Zhou, Y., Prakash, P. S., Liang, H., Gorfe, A. A. & Hancock, J. F. The KRAS and other prenylated polybasic domain membrane anchors recognize phosphatidylserine acyl chain structure. *Proc. Natl. Acad. Sci. USA* **118**, 10.1073/pnas.2014605118 (2021). 10.1073/pnas.2014605118 PMC8017956 33526670

[35] Zhou, Y. et al. Lipid-sorting specificity encoded in K-Ras membrane anchor regulates signal output. *Cell* **168**, 239–251 e216 (2017). 28041850 10.1016/j.cell.2016.11.059 PMC5653213

[36] van der Hoeven, D. et al. Sphingomyelin metabolism is a regulator of K-Ras function. *Mol. Cell Biol.* **38**, 10.1128/MCB.00373-17 (2018). 10.1128/MCB.00373-17 PMC5770534 29158292

[37] Kattan, W. E. et al. Components of the phosphatidylserine endoplasmic reticulum to plasma membrane transport mechanism as targets for KRAS inhibition in pancreatic cancer. *Proc. Natl. Acad. Sci. USA* **118**, 10.1073/pnas.2114126118 (2021). 10.1073/pnas.2114126118 PMC8713765 34903667

[38] Liu, J. et al. Glycolysis regulates KRAS plasma membrane localization and function through defined glycosphingolipids. *Nat. Commun.* **14**, 465 (2023). 36709325 10.1038/s41467-023-36128-5 PMC9884228

[39] Arora, N., Mu, H., Liang, H., Zhao, W. & Zhou, Y. RAS G-domains allosterically contribute to the recognition of lipid headgroups and acyl chains. *J. Cell Biol.* **223**, 10.1083/jcb.202307121 (2024). 10.1083/jcb.202307121 PMC10857904 38334958

[40] Morstein, J. et al. Direct modulators of K-Ras-membrane interactions. *ACS Chem. Biol.* **18**, 2082–2093 (2023). 37579045 10.1021/acschembio.3c00413 PMC10510109

[41] Lobell, R. B. et al. Evaluation of farnesyl:protein transferase and geranylgeranyl:protein transferase inhibitor combinations in preclinical models. *Cancer Res.* **61**, 8758–8768 (2001). 11751396

[42] Marom, M. et al. Selective inhibition of Ras-dependent cell growth by farnesylthiosalisylic acid. *J. Biol. Chem.* **270**, 22263–22270 (1995). 7673206 10.1074/jbc.270.38.22263

[43] Riely, G. J. et al. A phase II trial of Salirasib in patients with lung adenocarcinomas with KRAS mutations. *J. Thorac. Oncol.* **6**, 1435–1437 (2011). 21847063 10.1097/JTO.0b013e318223c099

[44] Schmick, M., Kraemer, A. & Bastiaens, P. I. Ras moves to stay in place. *Trends Cell Biol.* **25**, 190–197 (2015). 25759176 10.1016/j.tcb.2015.02.004

[45] Brandt, A. C., Koehn, O. J. & Williams, C. L. SmgGDS: an emerging master regulator of prenylation and trafficking by small GTPases in the Ras and Rho families. *Front. Mol. Biosci.* **8**, 685135 (2021). 34222337 10.3389/fmolb.2021.685135 PMC8242357

[46] Chandra, A. et al. The GDI-like solubilizing factor PDEdelta sustains the spatial organization and signalling of Ras family proteins. *Nat. Cell Biol.* **14**, 148–158 (2011). 22179043 10.1038/ncb2394

[47] Grant, B. M. M. et al. Calmodulin disrupts plasma membrane localization of farnesylated KRAS4b by sequestering its lipid moiety. *Sci. Signal.* **13**, 10.1126/scisignal.aaz0344 (2020). 10.1126/scisignal.aaz0344 32234958

[48] Sperlich, B., Kapoor, S., Waldmann, H., Winter, R. & Weise, K. Regulation of K-Ras4B membrane binding by calmodulin. *Biophys. J.* **111**, 113–122 (2016). 27410739 10.1016/j.bpj.2016.05.042 PMC4945620

[49] Zhou, M. et al. VPS35 binds farnesylated N-Ras in the cytosol to regulate N-Ras trafficking. *J. Cell Biol.* **214**, 445–458 (2016). 27502489 10.1083/jcb.201604061 PMC4987297

[50] Dharmaiah, S. et al. Structural basis of recognition of farnesylated and methylated KRAS4b by PDEd *d**elta*. *Proc. Natl. Acad. Sci. USA* **113**, E6766–E6775 (2016). 27791178 10.1073/pnas.1615316113 PMC5098621

[51] Fansa, E. K., Kosling, S. K., Zent, E., Wittinghofer, A. & Ismail, S. PDE6delta-mediated sorting of INPP5E into the cilium is determined by cargo-carrier affinity. *Nat. Commun.* **7**, 11366 (2016). 27063844 10.1038/ncomms11366 PMC5512577

[52] Grant, B. M. M., Enomoto, M., Ikura, M. & Marshall, C. B. A non-canonical calmodulin target motif comprising a polybasic region and lipidated terminal residue regulates localization. *Int. J. Mol. Sci.* **21**, 10.3390/ijms21082751 (2020). 10.3390/ijms21082751 PMC7216078 32326637

[53] Alvarez-Moya, B., Lopez-Alcala, C., Drosten, M., Bachs, O. & Agell, N. K-Ras4B phosphorylation at Ser181 is inhibited by calmodulin and modulates K-Ras activity and function. *Oncogene* **29**, 5911–5922 (2010). 20802526 10.1038/onc.2010.298

[54] Cho, K. J. et al. AMPK and endothelial nitric oxide synthase signaling regulates K-Ras plasma membrane interactions via Cyclic GMP-dependent protein kinase 2. *Mol. Cell Biol.* **36**, 3086–3099 (2016). 27697864 10.1128/MCB.00365-16 PMC5126295

[55] Kaya, P. et al. An improved PDE6D inhibitor combines with sildenafil to inhibit KRAS mutant cancer cell growth. *J. Med. Chem.* **67**, 8569–8584 (2024). 38758695 10.1021/acs.jmedchem.3c02129 PMC11181323

[56] Hait, W. N. et al. The effect of calmodulin inhibitors with bleomycin on the treatment of patients with high-grade gliomas. *Cancer Res.* **50**, 6636–6640 (1990). 1698540

[57] Manoharan, G. B., Kopra, K., Eskonen, V., Harma, H. & Abankwa, D. High-throughput amenable fluorescence-assays to screen for calmodulin-inhibitors. *Anal. Biochem.* **572**, 25–32 (2019). 30825429 10.1016/j.ab.2019.02.015

[58] Okutachi, S. et al. A covalent calmodulin inhibitor as a tool to study cellular mechanisms of K-Ras-driven stemness. *Front. Cell Dev. Biol.* **9**, 665673 (2021). 34307350 10.3389/fcell.2021.665673 PMC8296985

[59] Manoharan, G. B., Laurini, C., Bottone, S., Ben Fredj, N. & Abankwa, D. K. K-Ras binds calmodulin-related centrin1 with potential implications for K-Ras driven cancer cell stemness. *Cancers* **15**, 10.3390/cancers15123087 (2023). 10.3390/cancers15123087 PMC10296094 37370697

[60] Abraham, S. J., Nolet, R. P., Calvert, R. J., Anderson, L. M. & Gaponenko, V. The hypervariable region of K-Ras4B is responsible for its specific interactions with calmodulin. *Biochemistry* **48**, 7575–7583 (2009). 19583261 10.1021/bi900769j PMC2729490

[61] Villalonga, P. et al. Calmodulin binds to K-Ras, but not to H- or N-Ras, and modulates its downstream signaling. *Mol. Cell Biol.* **21**, 7345–7354 (2001). 11585916 10.1128/MCB.21.21.7345-7354.2001 PMC99908

[62] Li, C. J. et al. Dynamic redistribution of calmodulin in HeLa cells during cell division as revealed by a GFP-calmodulin fusion protein technique. *J. Cell Sci.* **112**, 1567–1577 (1999). 10212150 10.1242/jcs.112.10.1567

[63] Yu, Y. Y. et al. The association of calmodulin with central spindle regulates the initiation of cytokinesis in HeLa cells. *Int. J. Biochem. Cell Biol.* **36**, 1562–1572 (2004). 15147735 10.1016/j.biocel.2003.12.016

[64] Martin-Gago, P., Fansa, E. K., Wittinghofer, A. & Waldmann, H. Structure-based development of PDEdelta inhibitors. *Biol. Chem.* **398**, 535–545 (2017). 27935847 10.1515/hsz-2016-0272

[65] Siddiqui, F. A. et al. PDE6D inhibitors with a new design principle selectively block K-Ras activity. *ACS Omega* **5**, 832–842 (2020). 31956834 10.1021/acsomega.9b03639 PMC6964506

[66] Fansa, E. K. & Wittinghofer, A. Sorting of lipidated cargo by the Arl2/Arl3 system. *Small GTPases* **7**, 222–230 (2016). 27806215 10.1080/21541248.2016.1224454 PMC5129900

[67] Lauth, M. et al. DYRK1B-dependent autocrine-to-paracrine shift of Hedgehog signaling by mutant RAS. *Nat. Struct. Mol. Biol.* **17**, 718–725 (2010). 20512148 10.1038/nsmb.1833

[68] Yelland, T. et al. Stabilization of the RAS:PDE6D complex is a novel strategy to inhibit RAS signaling. *J. Med. Chem.* **65**, 1898–1914 (2022). 35104933 10.1021/acs.jmedchem.1c01265 PMC8842248

[69] Chen, C. & Yamashita, Y. M. Centrosome-centric view of asymmetric stem cell division. *Open Biol.* **11**, 200314 (2021). 33435817 10.1098/rsob.200314 PMC7881172

[70] Thomas, S. et al. A homozygous PDE6D mutation in Joubert syndrome impairs targeting of farnesylated INPP5E protein to the primary cilium. *Hum. Mutat.* **35**, 137–146 (2014). 24166846 10.1002/humu.22470 PMC3946372

[71] Arora, C. et al. The landscape of cancer-rewired GPCR signaling axes. *Cell Genom.* **4**, 100557 (2024). 38723607 10.1016/j.xgen.2024.100557 PMC11099383

[72] Wang, F. et al. Gliotransmission and adenosine signaling promote axon regeneration. *Dev. Cell* **58**, 660–676 e667 (2023). 37028426 10.1016/j.devcel.2023.03.007 PMC10173126

[73] Patterson, V. L. & Burdine, R. D. Swimming toward solutions: using fish and frogs as models for understanding RASopathies. *Birth Defects Res.* **112**, 749–765 (2020). 32506834 10.1002/bdr2.1707 PMC7968373

[74] Palla, A. R. et al. Primary cilia on muscle stem cells are critical to maintain regenerative capacity and are lost during aging. *Nat. Commun.* **13**, 1439 (2022). 35301320 10.1038/s41467-022-29150-6 PMC8931095

[75] Yohe, M. E. et al. MEK inhibition induces MYOG and remodels super-enhancers in RAS-driven rhabdomyosarcoma. *Sci. Transl. Med.* **10**, 10.1126/scitranslmed.aan4470 (2018). 10.1126/scitranslmed.aan4470 PMC8054766 29973406

[76] Dajee, M., Tarutani, M., Deng, H., Cai, T. & Khavari, P. A. Epidermal Ras blockade demonstrates spatially localized Ras promotion of proliferation and inhibition of differentiation. *Oncogene* **21**, 1527–1538 (2002). 11896581 10.1038/sj.onc.1205287

