# Supplementary material for: Drugging Ras trafficking—are there new roads to travel?
Source: NPJ Drug Discov. 2025 Jun 4;2:9. doi: 10.1038/s44386-025-00012-7 (PMC13267117; doi:10.1038/s44386-025-00012-7)
Supplement: Supplementary file 1 — SI Tables [file 44386_2025_12_MOESM1_ESM.pdf]

Supplementary Information

Drugging Ras Trafficking – Are there new roads to travel?

Elisabeth Schaffner-Reckinger #, Atanasio Gómez-Mulas # and Daniel Kwaku Abankwa*

Cancer Cell Biology and Drug Discovery Group, Department of Life Sciences and Medicine, University of Luxembourg, 4362 Esch-sur-Alzette, Luxembourg

# These authors contributed equally
* Correspondence: daniel.abankwa@uni.lu

Table S1. Overview of several direct Ras inhibitors under development.

Inhibitor	Target	IC_{50} (nM)	Binding site	Clinical phase	Reference
**KRASG12C inhibitors**					
AMG-510 [Sotorasib]	KRASG12C (OFF)	pERK: 30<br>Cell viability: 9-6	Switch II pocket	FDA approved	1
MRTX849 [Adagrasib]	KRASG12C (OFF)	pERK: 16.3-4.7<br>Cell viability: <100	Switch II pocket	FDA approved	2
D-1553 [Garsorasib]	KRASG12C (OFF)	pERK: 6.9<br>Cell viability: <33	Switch II pocket	NCT04585035<br>NCT05383898	3
JDQ443 [Opnurasib]	KRASG12C (OFF)	pERK: <63<br>Cell viability: <133	Switch II pocket	NCT05445843<br>NCT04699188<br>NCT05358249	4
LY3537982 [Olomorasib]	KRASG12C (OFF)	pERK: 0.65	Not specified	NCT04956640	5
GDC-6036 [Divarasib]	KRASG12C (OFF)	Cell viability: 0.18	Switch II pocket	NCT04449874	6
JAB-21822 [Glecirasib]	RASG12C (OFF)	Cell viability: <100	Switch II pocket	NCT05009329<br>NCT05002270<br>NCT05288205	7
GFH925 [Fulzerasib]	KRASG12C (OFF)	pERK: 37<br>Cell viability: 20-2	Not specified	NCT05005234	8
BI 1823911	KRASG12C (OFF)	Cell viability: <100	Not specified	NCT04973163	9
RMC-6291	KRASG12C (ON)	pERK: 0.7<br>Cell viability: 0.11	CypA:compound:RAS tricomplex	NCT05462717<br>NCT06162221<br>NCT06128551	10
**KRASG12D inhibitors**					
MRTX1133	KRASG12D (OFF)	pERK: <10<br>Cell viability: 6	Switch II pocket	NCT05737706	11
(R)-G12Di-7	KRASG12D (ON/ OFF)	Cell viability: 73	Switch II pocket	NA	12
RMC-9805	KRASG12D (ON)	pERK: 7<br>Cell viability: 23	CypA:compound:RAS tri-complex	NCT06040541	13
**Other KRAS mutant inhibitors**					
RMC-0708 (RM-046)	KRASQ61H (ON)	NA	CypA:compound:RAS tri-complex	IND-enabling	14
RMC-5127	KRASG12V (ON)	pERK: 0.6<br>Cell viability: 2.1	CypA:compound:RAS tri-complex	IND-enabling	15
RMC-8839	KRASG13C (ON)	pERK: 0.4<br>Cell viability: 1.1	CypA:compound:RAS tri-complex	IND-enabling	16
**pan-RAS inhibitors**					
BI-2865	pan-KRAS (OFF)	Cell viability: 140	Switch II pocket	NA	17
YK-8S	KRASG12C/D (ON/ OFF)	Cell viability: <6.7	Switch II pocket	NA	18
RMC-6236	pan-RAS (ON)	pERK: <2.14<br>Cell viability: <1.4	CypA:compound:RAS tri-complex	NCT05379985<br>NCT06162221	19

References Table S1

1 Canon, J. *et al.* The clinical KRAS(G12C) inhibitor AMG 510 drives anti-tumour immunity. *Nature* **575**, 217-223 (2019). https://doi.org/10.1038/s41586-019-1694-1

2 Hallin, J. *et al.* The KRASG12C Inhibitor MRTX849 Provides Insight toward Therapeutic Susceptibility of KRAS-Mutant Cancers in Mouse Models and Patients. *Cancer Discovery* **10**, 54-71 (2020). https://doi.org/10.1158/2159-8290.Cd-19-1167

3 Shi, Z. *et al.* D-1553: A novel KRAS(G12C) inhibitor with potent and selective cellular and in vivo antitumor activity. *Cancer Sci* **114**, 2951-2960 (2023). https://doi.org/10.1111/cas.15829

4 Weiss, A. *et al.* Discovery, Preclinical Characterization, and Early Clinical Activity of JDQ443, a Structurally Novel, Potent, and Selective Covalent Oral Inhibitor of KRASG12C. *Cancer Discov* **12**, 1500-1517 (2022). https://doi.org/10.1158/2159-8290.Cd-22-0158

5 Peng, S.-B. *et al.* Abstract 1259: Preclinical characterization of LY3537982, a novel, highly selective and potent KRAS-G12C inhibitor. *Cancer Research* **81**, 1259-1259 (2021). https://doi.org/10.1158/1538-7445.Am2021-1259

6 Purkey, H. in *AACR Annual Meeting 2022* Vol. 82 (Cancer Res, New Orleans, 2022).

7 Li, J. *et al.* Preliminary activity and safety results of KRAS G12C inhibitor glecirasib (JAB-21822) in patients with pancreatic cancer and other solid tumors. *Journal of Clinical Oncology* **42**, 604-604 (2024). https://doi.org/10.1200/JCO.2024.42.3_suppl.604

8 Rosell, R. *et al.* KRAS G12C-mutant driven non-small cell lung cancer (NSCLC). *Critical Reviews in Oncology/Hematology* **195**, 104228 (2024). https://doi.org/https://doi.org/10.1016/j.critrevonc.2023.104228

9 Waizenegger, I. C. *et al.* Abstract 2667: Trial in progress: Phase 1 study of BI 1823911, an irreversible KRASG12C inhibitor targeting KRAS in its GDP-loaded state, as monotherapy and in combination with the pan-KRAS SOS1 inhibitor BI 1701963 in solid tumors expressing KRASG12C mutation. *Cancer Research* **82**, 2667-2667 (2022). https://doi.org/10.1158/1538-7445.Am2022-2667

10 Schulze, C. J. *et al.* Chemical remodeling of a cellular chaperone to target the active state of mutant KRAS. *Science* **381**, 794-799 (2023). https://doi.org/10.1126/science.adg9652

11 Wang, X. *et al.* Identification of MRTX1133, a Noncovalent, Potent, and Selective KRASG12D Inhibitor. *Journal of Medicinal Chemistry* **65**, 3123-3133 (2022). https://doi.org/10.1021/acs.jmedchem.1c01688

12 Zheng, Q., Zhang, Z., Guiley, K. Z. & Shokat, K. M. Strain-release alkylation of Asp12 enables mutant selective targeting of K-Ras-G12D. *Nat Chem Biol* **20**, 1114-1122 (2024). https://doi.org/10.1038/s41589-024-01565-w

13 Menard, M. J. *et al.* Abstract 3475: RMC-9805, a first-in-class, mutant-selective, covalent and orally bioavailable KRASG12D(ON) inhibitor, promotes cancer-associated neoantigen recognition and synergizes with immunotherapy in preclinical models. *Cancer Research* **83**, 3475-3475 (2023). https://doi.org/10.1158/1538-7445.Am2023-3475

14 Yang, Y. C. *et al.* Abstract 1598: RM-046, a first-in-class, mutant-selective, and oral KRASQ61H(ON) inhibitor that drives tumor regression in preclinical models and validates KRASQ61H as a therapeutic target. *Cancer Research* **83**, 1598-1598 (2023). https://doi.org/10.1158/1538-7445.Am2023-1598

15 Chen, Z. *et al.* Abstract 3340: RMC-5127, a first-in-class, orally bioavailable mutant-selective RASG12V(ON) inhibitor is central nervous system (CNS)-penetrant and demonstrates anti-tumor activity in a preclinical intracranial xenograft model. *Cancer Research* **84**, 3340-3340 (2024). https://doi.org/10.1158/1538-7445.Am2024-3340

16 Schulze, C. J. *et al.* Abstract 3598: A first-in-class tri-complex KRASG13C(ON) inhibitor validates therapeutic targeting of KRASG13Cand drives tumor regressions in preclinical models. *Cancer Research* **82**, 3598-3598 (2022). https://doi.org/10.1158/1538-7445.Am2022-3598

17 Kim, D. *et al.* Pan-KRAS inhibitor disables oncogenic signalling and tumour growth. *Nature* **619**, 160-166 (2023). https://doi.org/10.1038/s41586-023-06123-3

18 Yu, Z. *et al.* Simultaneous Covalent Modification of K-Ras(G12D) and K-Ras(G12C) with Tunable Oxirane Electrophiles. *J Am Chem Soc* **145**, 20403-20411 (2023). https://doi.org/10.1021/jacs.3c05899

19 Jiang, J. *et al.* Translational and Therapeutic Evaluation of RAS-GTP Inhibition by RMC-6236 in RAS-Driven Cancers. *Cancer Discov* **14**, 994-1017 (2024). https://doi.org/10.1158/2159-8290.Cd-24-0027

Table S2. Overview of current direct PDE6D inhibitors. Abbreviations for assays used to determine the K_D: FP- fluorescence polarization; SPR- surface plasmon resonance spectroscopy; ITC- isothermal titration calorimetry.

Inhibitor	K_D	Structure	Reference
Deltarasin	38 ± 16 nM (FP)	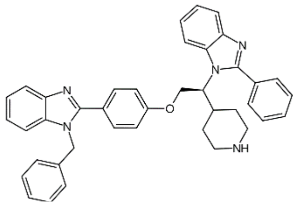	1
Deltazinone1	8 ± 4 nM (FP)	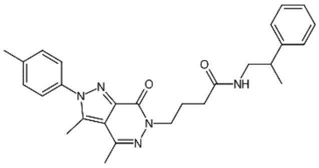	2
Deltasonamide1	203 ± 31 pM (FP)	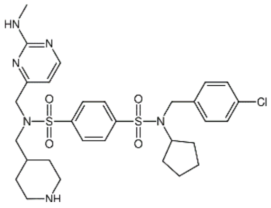	3
Compound 99	8 ± 4 nM (FP)	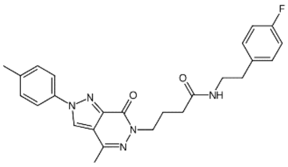	4
Deltaflexin-2	2.92 ± 0.02 µM (SPR)	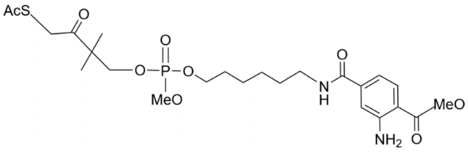	5
Deltaflexin3	6 ± 1 nM (FP)	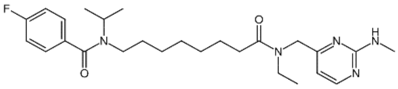	6
DW0254	436 ± 6 nM (ITC)	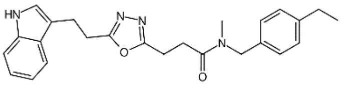	7
Compound 3b	2.0 ± 0.5 nM (FP)	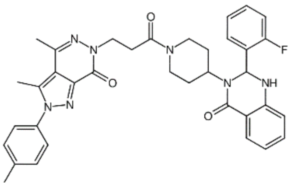	8

Inhibitor	K_D	Structure	Reference
Compound PD3	0.5 ± 0.2 μM (FP)	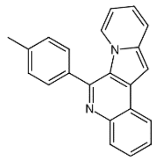	9
Compound 11b	38 ± 17 nM (FP)	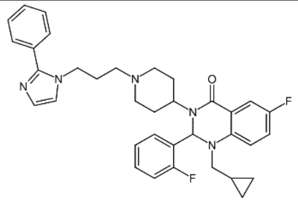	10
Compound 36l	127 ± 16 nM (FP)	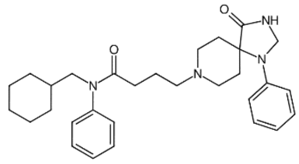	11

Degrader	K_D	Structure	Reference
Compound 3	64 ± 2 nM (FP)	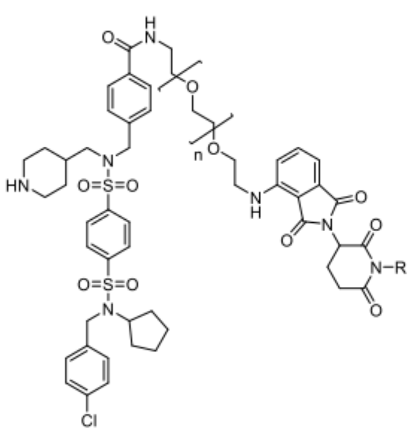 n=3, R=H	12
Compound 5	57 ± 2 nM (FP)	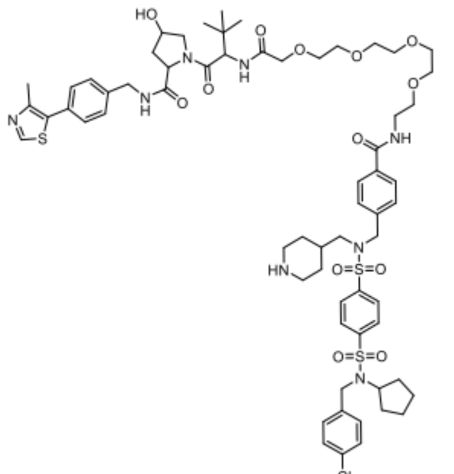	12

Degrader	K_D	Structure	Reference
Compound 17f	9.0 ± 1.3 nM (FP)	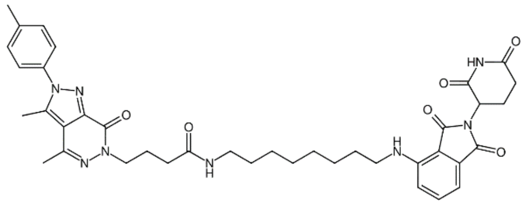	13

References Table S2

1 Zimmermann, G. *et al.* Small molecule inhibition of the KRAS-PDEdelta interaction impairs oncogenic KRAS signalling. *Nature* **497**, 638-642 (2013). https://doi.org/10.1038/nature12205
2 Papke, B. *et al.* Identification of pyrazolopyridazinones as PDEdelta inhibitors. *Nat Commun* **7**, 11360 (2016). https://doi.org/10.1038/ncomms11360
3 Martin-Gago, P. *et al.* A PDE6delta-KRas Inhibitor Chemotype with up to Seven H-Bonds and Picomolar Affinity that Prevents Efficient Inhibitor Release by Arl2. *Angew Chem Int Ed Engl* **56**, 2423-2428 (2017). https://doi.org/10.1002/anie.201610957
4 Murarka, S. *et al.* Development of Pyridazinone Chemotypes Targeting the PDEdelta Prenyl Binding Site. *Chemistry* **23**, 6083-6093 (2017). https://doi.org/10.1002/chem.201603222
5 Siddiqui, F. A. *et al.* PDE6D Inhibitors with a New Design Principle Selectively Block K-Ras Activity. *ACS Omega* **5**, 832-842 (2020). https://doi.org/10.1021/acsomega.9b03639
6 Kaya, P. *et al.* An Improved PDE6D Inhibitor Combines with Sildenafil To Inhibit KRAS Mutant Cancer Cell Growth. *J Med Chem* **67**, 8569-8584 (2024). https://doi.org/10.1021/acs.jmedchem.3c02129
7 Canovas Nunes, S. *et al.* Validation of a small molecule inhibitor of PDE6D-RAS interaction with favorable anti-leukemic effects. *Blood Cancer Journal* **12**, 64 (2022). https://doi.org/10.1038/s41408-022-00663-z
8 Jiang, Y. *et al.* Structural Biology-Inspired Discovery of Novel KRAS-PDEdelta Inhibitors. *J Med Chem* **60**, 9400-9406 (2017). https://doi.org/10.1021/acs.jmedchem.7b01243
9 Lee, J. *et al.* Development of PD3 and PD3-B for PDEdelta inhibition to modulate KRAS activity. *J Enzyme Inhib Med Chem* **37**, 1656-1666 (2022). https://doi.org/10.1080/14756366.2022.2086865
10 Chen, L., Zhuang, C., Lu, J., Jiang, Y. & Sheng, C. Discovery of Novel KRAS-PDEdelta Inhibitors by Fragment-Based Drug Design. *J Med Chem* **61**, 2604-2610 (2018). https://doi.org/10.1021/acs.jmedchem.8b00057
11 Chen, L. *et al.* Discovery of novel KRAS–PDEdelta inhibitors with potent activity in patient-derived human pancreatic tumor xenograft models. *Acta Pharm Sin B* **12**, 274-290 (2022). https://doi.org/10.1016/j.apsb.2021.07.009
12 Winzker, M. *et al.* Development of a PDEdelta-Targeting PROTACs that Impair Lipid Metabolism. *Angew Chem Int Ed Engl* **59**, 5595-5601 (2020). https://doi.org/10.1002/anie.201913904
13 Cheng, J., Li, Y., Wang, X., Dong, G. & Sheng, C. Discovery of Novel PDEdelta Degraders for the Treatment of KRAS Mutant Colorectal Cancer. *J Med Chem* **63**, 7892-7905 (2020). https://doi.org/10.1021/acs.jmedchem.0c00929
